# Simultaneous spectrophotometric determination of copper(II), lead(II) and cadrnium(II)

**DOI:** 10.1155/S1463924695000204

**Published:** 1995

**Authors:** Shouxin Ren, Ling Gao

**Affiliations:** Department of Chemistry Inner Mongolian University Inner Mongolia Huhehot 010021 China

## Abstract

The full spectrum technique in the fourier domain was studied for simultaneous determination of Cu(II), Pb(II) and Cd(II) with 4-(2-pyridylazo) resorcinol (PAR). A program called SPGRFSQ. was designed to perform the calculations. Seven error functions were calculated for deducing the number of factors. Data reduction was performed using principal component analysis (PCA) algorithm. Experimental results showed the method to be successful even where there was severe overlap of spectra.


Journal of Automatic Chemistry, Vol. 17, No. 3 (May-June 1995), pp. 115-118

### Simultaneous spectrophotometric determination of copper(II), lead(II) and cadmium(II)

**Shouxin Ren and Ling Gao**
*Department of Chemistry, Inner Mongolian University, 010021 Huhehot, Inner Mongolia, China*

*The full spectrum technique in the fourier domain was studied for simultaneous determination of Cu(II), Pb(II) and Cd(II) with 4-(2-pyridylazo) resorcinol (PAR). A program called SPGRFSQ was designed to perform the calculations. Seven error functions were calculated for deducing the number of factors. Data reduction was performed using principal component analysis (PCA) algorithm. Experimental results showed the method to be successful even where there was severe overlap of spectra.*

### Introduction

The development of chemometric methods of multicomponent analysis has allowed the resolution of the complex spectra of mixtures of analytes. Various research groups have reported using chemometric methods to resolve overlapping spectra [1, 2]. Multicomponent determinations have recently been improved by using the full spectrum method and different chemometric algorithms [3, 4].

Simultaneous determination of copper(II), lead(II) and cadmium(II) with 4-(2-pyridylazo) resorcinol (PAR) using traditional spectrophotometry is difficult because (1) the absorption spectra overlap; and (2) the superimposed curve is not suitable for quantitative evaluation. In a PAR system, in addition to low selectivity, problems arise because of strong absorbance of the reagent. This paper shows that multicomponent analysis with full spectrum methods is a solution.

### Experimental

### *Instruments and apparatus*

The Shimadzu UV-265 and UV-120 spectrophotometers were used for all experiments; a GW286 EX/16 microcomputer with a maths coprocessor was used for the calculations; and a Mettler DL 21 titrator was used for standardization of the standard solutions.

### *Reagents*

All reagents were of analytical reagent grade. The water used was doubly distilled and deionized. Stock standard solutions of Cu(II), Pb(II) and Cd(II) were prepared from nitrates and standardized titrimetrically with EDTA. Buffer solution (pH 7·0) was prepared from borax solution and hydrochloric acid solution; a 0·02% m/v PAR solution in alcohol was used.

### *Procedure*

A series of mixed standard solutions containing various ratios of the three metal ions at pH 7·0 was prepared in 50 ml standard flasks, 1 ml 0·02% m/v PAR solution in alcohol, 15 ml of buffer solution (pH 7·0) and dilution with distilled water to mark. Cuvettes with a path length of 1 cm were used and the blank absorbance due to PAR absorption was subtracted. Absorbances were measured between 460 nm and 560 nm at 5 and 2 nm distances, giving values at 33 wavelengths for each standard solution. An absorption matrix, $D$, was built up from these data.

### *Computer program and algorithms*

The $D$ matrix, obtained as described above, is transformed using fast fourier transform (FFT) to produce the $F$ matrix. The variance-covariance matrix $A$ ($FF^1$) can be subjected to singular value decomposition for calculation of the eigenvalues and eigenvectors. The number of species is estimated by several criteria, which are based on the theory of error in factor analysis [5-7]. After determining the number of species ($t$), the set of $t + 2$ significant eigenvectors becomes the basis eigenvectors ($\mathbf{U}$). The projection of each standard mixture is calculated by $M = \mathbf{U}^T F$. A proportionality matrix, $P$, is found by least squares regression analysis, $P = C_k M^T (MM^T)^{-1}$. The absorbance spectra for unknown mixtures is converted to the fourier domain and represented as $Fu$. The projection matrix ($Mu$) is obtained by matrix multiplication: $Mu = \mathbf{U}^T Fu$. The projection matrix ($Mu$) is then multiplied by $P$ to give the unknown concentration as $Cu = PMu$. A program called SPGRFSQ, which is based on the algorithm, was designed to perform the calculation.

### Results and discussion

### *Determination of the number of factors*

Seven criteria were used to calculate the number of factors—see table 1. Results are shown in table 2. The minimum of the Frac function appeared at $t = 4$: the appropriate number of eigenvectors is one less than that giving the minimum frac value. Figure 1 shows the Frac function plotted against the number of factors; the number of factors was 3. The magnitude of REV decreased rapidly until $t = 3$, then it stabilized. The IND function reached a minimum at 2-4; the magnitude of the first four eigenvalues were larger than those of 5-8; and a maximum of the eigenvalue ratio (ER) function appeared at 4. The discrepancy in these results was probably due to the existence of some other weakly absorbing species. The speciation of the system was very complex. The results

0142-0453/95 $10.00 © 1995 Taylor & Francis Ltd.

*Table 1. Factor analysis criteria.*

| Name | Formulae | Name | Formulae |
|---|---|---|---|
| Rear error | $RE = \left[\frac{\sum_{j=n+1}^{k} \lambda_j}{L(k-n)}\right]^{1/2}$ | Indication function | $IND = RE/(k-n)^2$ |
| Extracted error | $XE = RE\left(\frac{k-n}{k}\right)^{1/2}$ | Eigenvalue ratio | $ER_j = \lambda_j/\lambda_{j+1}$ |
| Imbedded error | $IE = RE\left(\frac{n}{k}\right)^{1/2}$ | Reduced eigenvalue | $REV_j = \frac{\lambda_j}{(L-j+1)(k-j+1)}$ |
| Fraction function | $FRAC = \frac{\lambda_j}{\sum_{j=1}^{k} \lambda_j}$ | | |

*Table 2. Results on factor analysis for the Cu(II)/Pb(II)/Cd(II)/PAR systems.*

| $t$ | EV ×1·0E−3 | RE | IND | XE | IE | ER | REV | Frac |
|---|---|---|---|---|---|---|---|---|
| 1 | 2·0305 | 0·0926 | 0·0019 | 0·0866 | 0·0327 | 556·5122 | 3·9658 | 0·9981 |
| 2 | 0·0036 | 0·0224 | 0·0006 | 0·0194 | 0·0112 | 33·7469 | 3·0083 | 0·0018 |
| 3 | 0·0001 | 0·0162 | 0·0006 | 0·0128 | 0·0099 | 1·8777 | 0·0003 | 0·0001 |
| 4 | 0·0001 | 0·0102 | 0·0006 | 0·0072 | 0·0072 | 3·6458 | 0·0002 | 0·0000 |
| 5 | 0·0000 | 0·0075 | 0·0006 | 0·0046 | 0·0060 | 1·7198 | 0·0001 | 0·0000 |
| 6 | 0·0000 | 0·0037 | 0·0008 | 0·0018 | 0·0032 | 8·5655 | 0·0001 | 0·0000 |
| 7 | 0·0000 | 0·0032 | 0·0009 | 0·0011 | 0·0030 | 1·6268 | 0·0000 | 0·0000 |
| 8 | 0·0000 | — | 0·0032 | — | — | — | 0·0000 | 0·0000 |

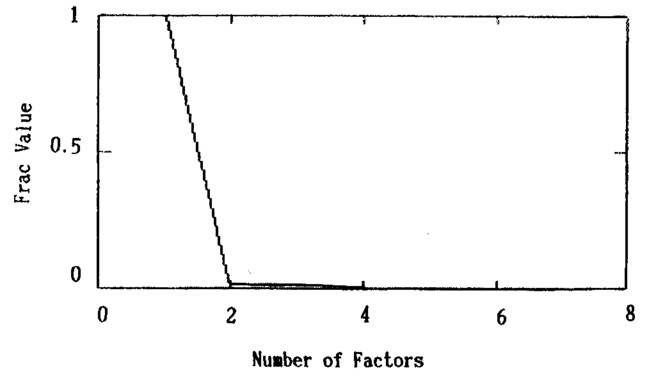

*Figure 1. Plot of the Frac function against factor number.*

indicated that three absorbing components may be present.

*The training set model*

Figure 2 shows the absorption spectra of Cu(II), Pb(II), Cd(II) and their mixed solution with PAR as reagent. The absorption maximums of Cu(II), Cd(II) and Pb(II) complexes were 507 nm, 498 nm and 512 nm, respectively. The concentrations of the three metal ions in eight standard solutions are shown in table 3. The spectra were measured between 460 nm and 560 nm at 5 and 2 nm distances, giving values at 33 wavelengths for every standard solution. Each of the spectra of the training set were corrected by subtracting the standard spectrum of reagent, then an absorption matrix, $D$, was built up.

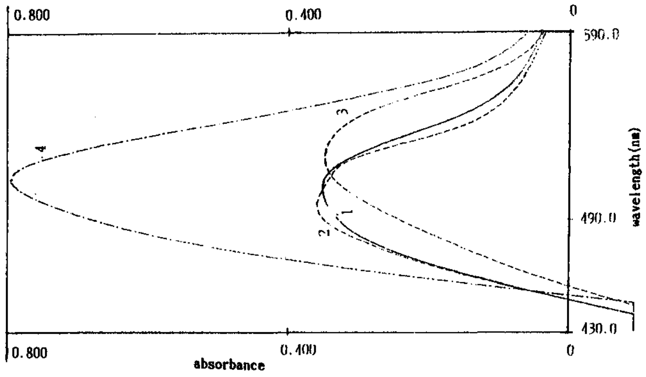

*Figure 2. Absorption spectra of Cu(II), Pb(II), Cd(II) and their mixed solution with PAR as reagent. 1: 1·2 × $10^{-5}$ mol/l Cu(II). 2: 1.2 × $10^{-5}$ mol/l Pb(II). 3: 1.2 × $10^{-5}$ mol/l Cd(II). 4: 1·2 × $10^{-5}$ mol/l Cu(II) + 1·2 × $10^{-5}$ mol/l Pb(II) + 1·6 × $10^{-5}$ mol/l Cd(II).*

Figure 3 is a three-dimensional plot of spectra of the training set obtained at 33 different wavelengths. Matrix $D$ was transformed with the use of a standard FFT principal. The fourier transform method also acts as a filter, since the high frequency noise terms can be deleted producing a significant reduction of error. The $F$ matrix preserved the important property of the Lambert–Beer relationship. The $P$ matrix is the calibration matrix and provides all the information that is needed to calculate the concentration of the unknown samples from the $Fu$

*Table 3. Composition of the standard solutions.*

| Solution number | Concentration (mmol/l) Cu(II) | Pb(II) | Cd(II) |
|---|---|---|---|
| 1 | 0·0165 | 0·9968 | 0·5869 |
| 2 | 0·8739 | 1·0134 | 0·6319 |
| 3 | 0·6427 | 0·8944 | 1·0318 |
| 4 | 1·0143 | 1·3534 | 0·9335 |
| 5 | 0·5233 | 0·7828 | 0·9203 |
| 6 | 0·4923 | 0·6448 | 0·5277 |
| 7 | 1·0975 | 1·0985 | 0·7155 |
| 8 | 0·7770 | 0·9470 | 0·9489 |

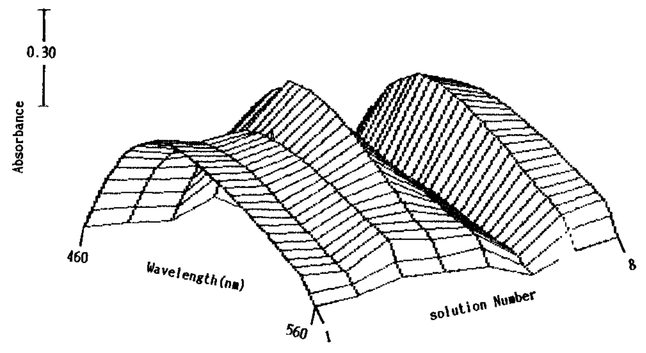

*Figure 3. Three-dimensional plot of the spectra in the training set.*

*Table 4. P matrix of the training set with five basis eigenvectors.*

| | | | | |
|---|---|---|---|---|
| 0·0156 | 0·1863 | 0·5264 | −0·1575 | −0·0272 |
| 0·0617 | 0·1065 | −0·3737 | −0·1638 | 0·0490 |
| 0·0497 | −0·2176 | −0·5462 | 0·2219 | 0·0217 |

matrix. The $P$ matrix was calculated using SPGRFSQ as shown in table 4.

*Data reduction*

Data reduction was based on the PCA algorithm. Using the SPGRFSQ program, the concentrations of 'unknowns' and the $P$ matrix were calculated while varying the number of basis eigenvectors. The results are given in table 5. Based on the results, it is recommended that five eigenvectors are used for analysis; eigenvectors above 6 should be ignored.

*Determination of unknowns*

The added concentrations of a set of eight synthetic 'unknown' samples are shown in table 6. The spectra of the unknown samples were measured in the same way as the training set model. Using SPGRFSQ the concentrations of the Cu(II), Pb(II) and Cd(II) were found and are given in table 7. Average recoveries and their relative deviations are listed in table 8. The experimental results showed that the full spectrum techniques in the fourier domain gave satisfactory results for simultaneous determination of a complex mixture with severely overlapping spectra.

*Table 5. Relationship between numbers of eigenvector and relative deviation of 'unknowns' determination.*

| Numbers of eigenvector | | Relative deviation (%) 'Unknowns' number (1) | (2) | (3) |
|---|---|---|---|---|
| 3 | Cu(II) | −0·0061 | −0·0172 | −0·0302 |
| | Pb(II) | −0·0031 | −0·0166 | −0·0313 |
| | Cd(II) | 0·0068 | 0·0706 | 0·0273 |
| 4 | Cu(II) | −0·0021 | −0·0022 | 0·0072 |
| | Pb(II) | 0·0004 | 0·0016 | −0·0065 |
| | Cd(II) | 0·0019 | 0·0069 | −0·0038 |
| 5 | Cu(II) | −0·0021 | −0·0018 | 0·0032 |
| | Pb(II) | 0·0003 | 0·0006 | −0·0020 |
| | Cd(II) | 0·0019 | 0·0057 | −0·0020 |

*Table 6. Composition of the unknown samples.*

| Species | Concentration (mmol/l) Sample number (1) | (2) | (3) | (4) | (5) | (6) | (7) | (8) |
|---|---|---|---|---|---|---|---|---|
| Cu(II) | 0·7987 | 0·8662 | 0·8711 | 0·6160 | 0·8352 | 0·7219 | 0·9599 | 0·4503 |
| Pb(H) | 1·1458 | 1·0021 | 0·9519 | 0·9419 | 1·0351 | 0·8781 | 0·8224 | 0·7062 |
| Cd(II) | 1·0008 | 0·8281 | 0·8569 | 0·8723 | 0·9162 | 0·8493 | 0·3176 | 0·7637 |

*Table 7. The concentrations of the unknowns calculated by SPGRFSQ.*

| Species | Concentration (mmol/l) Sample number (1) | (2) | (3) | (4) | (5) | (6) | (7) | (8) |
|---|---|---|---|---|---|---|---|---|
| Cu(II) | 0·8009 | 0·8653 | 0·8718 | 0·6164 | 0·8440 | 0·7204 | 0·9582 | 0·4518 |
| Pb(II) | 1·1448 | 1·0033 | 0·9555 | 0·9410 | 1·0367 | 0·8784 | 0·8229 | 0·7048 |
| Cd(II) | 0·9984 | 0·8289 | 0·8553 | 0·8708 | 0·9053 | 0·8509 | 0·3194 | 0·7622 |

The values were means of three replicate.

*Table 8. The average recoveries and their relative deviations of the 'unknowns'.*

| | Recovery (%) | | | | | | | |
|---|---|---|---|---|---|---|---|---|
| | Sample number | | | | | | | |
| Species | (1) | (2) | (3) | (4) | (5) | (6) | (7) | (8) |
| Cu(II) | 100·28 | 99·90 | 100·08 | 100·07 | 100·06 | 99·79 | 99·82 | 100·32 |
| Pb(II) | 99·92 | 100·12 | 100·38 | 99·90 | 100·15 | 100·03 | 100·06 | 99·80 |
| Cd(II) | 99·76 | 100·10 | 99·82 | 99·83 | 100·81 | 100·19 | 100·57 | 99·80 |
| | Relative deviation (%) | | | | | | | |
| | Sample number | | | | | | | |
| Species | (1) | (2) | (3) | (4) | (5) | (6) | (7) | (8) |
| Cd(II) | 0·0028 | −0·0011 | 0·0008 | 0·0007 | 0·0106 | −0·0021 | −0·0018 | 0·0032 |
| Pb(II) | −0·0008 | 0·0012 | 0·0038 | 0·0010 | −0·0015 | 0·0003 | 0·0006 | −0·0020 |
| Cd(II) | −0·0024 | 0·0010 | −0·0018 | −0·0017 | −0·0119 | 0·0019 | 0·0057 | −0·0020 |

The values were means of three replicate.

### *The condition number of the proportionality matrix P*

The condition number of the proportionality matrix $P$ (cond($P$)) indicates the numerical accuracy that can be lost when the matrix is inverted [8]. The condition number can be used as a measure for selectivity of the multicomponent system. The cond($P$) is usually calculated from the ratio of the largest eigenvalue of $P$ to the smallest eigenvalue; values for eigenvectors 3–7 were 11·9208, 11·4773, 11·4845, 11·4921 and 11·4961, respectively. As the number of eigenvectors increased, the value of cond($P$) remained almost constant—the selectivity of the spectrophotometric system did not change if the number of eigenvectors increased. This was due to the high degree of spectral overlap of the PAR complexes, giving the system low selectivity.

### Conclusion

Simultaneous determination of Cu(II), Pb(II) and Cd(II) with PAR by use of the full spectrum techniques in the fourier domain has been shown to be a successful method. The difficulty imposed by overlap of the absorption spectra was overcome by the method. Recoveries of Cu(II), Pb(II) and Cd(II) were between 98·8% and 101.1%.

### Acknowledgement

The authors would like to thank the National Natural Science Foundation of China for financial support.

### References

1. Beebe, K. R. and Kowalski, B. R., *Analytical Chemistry*, **59** (1987), 1007A.
2. Haaland, D. M. and Thomas, E. V., *Analytical Chemistry*, **60** (1988), 1193.
3. Marbach, R. and Heise, H. M., *Trends in Analytical Chemistry*, **11** (1992), 270.
4. Liang, Y., Kvalheim, O. M. and Manne, R., *Chemometrics and Intelligent Laboratory Systems*, **18** (1993), 235.
5. Malinowski, E. R., *Analytical Chemistry*, **49** (1977), 605.
6. Malinowski, E. R., *Journal of Chemometrics*, **1** (1987), 605.
7. Malinowski, E. R., *Journal of Chemometrics*, **3** (1988), 49.
8. Jochum, C., Jochum, P. and Kowalski, B. R., *Analytical Chemistry*, **53** (1982), 85.

